# The Relationship Between Fracture Area and Standing Pelvic Sagittal Inclination in Patients With Subchondral Fracture of the Femoral Head

**DOI:** 10.7759/cureus.94619

**Published:** 2025-10-15

**Authors:** Katsura Kagawa, Takuya Nakamura

**Affiliations:** 1 Orthopedic Surgery, Saiseikai Toyama Hospital, Toyama, JPN; 2 Orthopedic Surgery, Toyama Prefectural Central Hospital, Toyama, JPN; 3 Orthopedic Surgery, Toyama Nishi General Hospital, Toyama, JPN

**Keywords:** femoral head, fracture area, posterior pelvic tilt, spinopelvic harmony, standing position, subchondral fracture

## Abstract

Background: Some subchondral fractures of the femoral head follow a course of rapid destructive arthrosis (RDA) of the hip. It has also been suggested that there is an association between standing posterior pelvic tilt and the development of RDA. This study aimed to examine the relationship between fracture area and standing pelvic sagittal inclination in subchondral fractures of the femoral head.

Methods: This study included 27 patients with subchondral fractures of the femoral head. The fracture area (nine axial sections) and fracture width were observed on MRI. Patients with fractures of the anterior one-third of the femoral head were classified into the anterior (A) group, and patients with fractures not extending into the anterior area were designated the non-anterior (NA) group. The supine and standing center edge (CE) angles and standing sacral slope (SS) were measured on radiographs of the hip, and supine SS was measured on CT or MRI.

Results: There were 17 fractures in group A and 10 in group NA. In group A, standing SS - supine SS (ΔSS: -8.6°, range: -15.4° to -2.6°) and standing CE - supine CE (ΔCE: -7.1°, range: -18.6° to -2.1°) were significantly more negative than in group NA (-4.1°, range: -6.7° to -1.1°, p<0.01; and -2.8°, range: -6.3° to -0.1°, p=0.01). In group A, 11 patients had progression of collapse, and 13 patients underwent total hip arthroplasty. In group NA, there were no cases of progressive collapse, and joint preservation was possible in five patients. In patients with posterior pelvic tilt more than 8° in the supine to standing position, the fracture area was located anteriorly in all cases.

Conclusion: With subchondral fractures of the femoral head, there is a relationship between standing pelvic sagittal inclination posteriorly and fracture area, and this affects prognosis. Patients with fractures in the anterior superior portion of the femoral head have a poor prognosis.

## Introduction

Recently, it was reported that some subchondral fractures of the femoral head follow a course of rapid destructive arthrosis (RDA) of the hip [1-3]. In addition to age-related bone fragility, a decrease in the anterior coverage of the femoral head due to posterior pelvic tilt in the standing position has also been reported as a factor in the development of RDA [4,5]. The decrease in the anterior acetabular coverage of the femoral head is thought to cause a stress concentration in the anterior superior portion of the femoral head, which progresses to collapse of the femoral head and rapid destruction of the hip joint. However, we have not been able to find any reports showing a relationship between posterior pelvic tilt in the standing position and the occurrence of subchondral fractures of the femoral head. In the present study, we examined the relationship between fracture area and standing pelvic sagittal inclination in subchondral fractures of the femoral head.

## Materials and methods

Patients

Twenty-seven patients with subchondral fractures of the femoral head treated in our institution between December 2013 and February 2021 were included in this study. Patients were included if the joint line was preserved on radiographs at the initial visit and if magnetic resonance imaging (MRI) and standing radiographs of the hip joint were available. Patients who already had more than 2 mm of collapse of the femoral head or arthropathic changes of the hip joint at the initial examination were excluded. Six males and 21 females with a mean age of 68.2±11.2 years (range: 41-89 years) were included. The follow-up period was defined as the time from the initial visit to the time of symptom resolution or surgery.

Outcomes

The primary outcomes of this study were fracture area and pelvic sagittal inclination. Pelvic sagittal inclination was assessed by sacral slope (SS). Secondary outcomes were fracture width, acetabular coverage, presence of progressive collapse of the femoral head, and treatment.

MRI evaluations and radiological measurements

**Figure 1:**
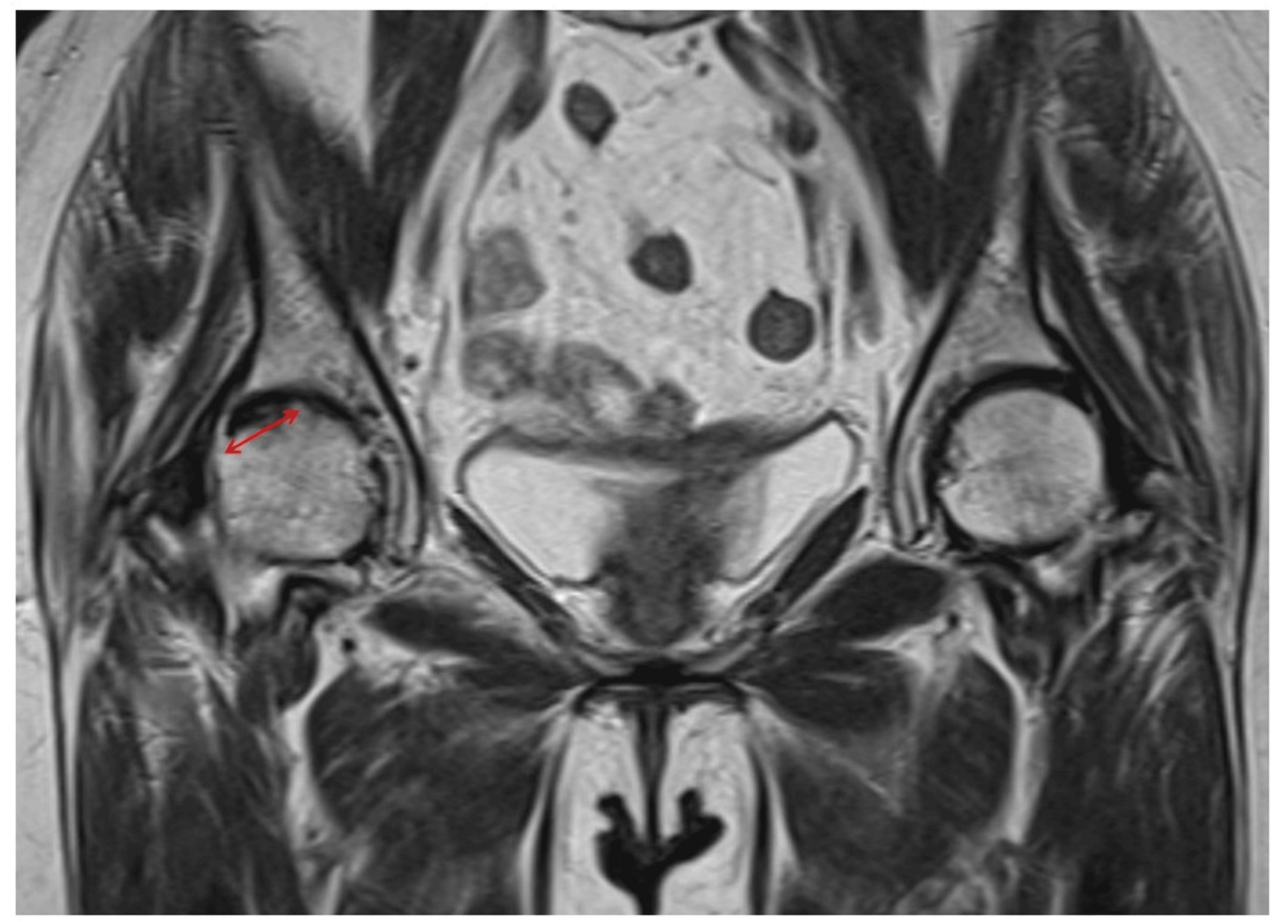
Measurement of fracture width on a T1-weighted coronal MRI view. The irregular, very low-intensity band was identified as the fracture line. The slice with the largest fracture area was selected, and the fracture width was measured. Double-headed arrow indicates the fracture width.

**Figure 2:**
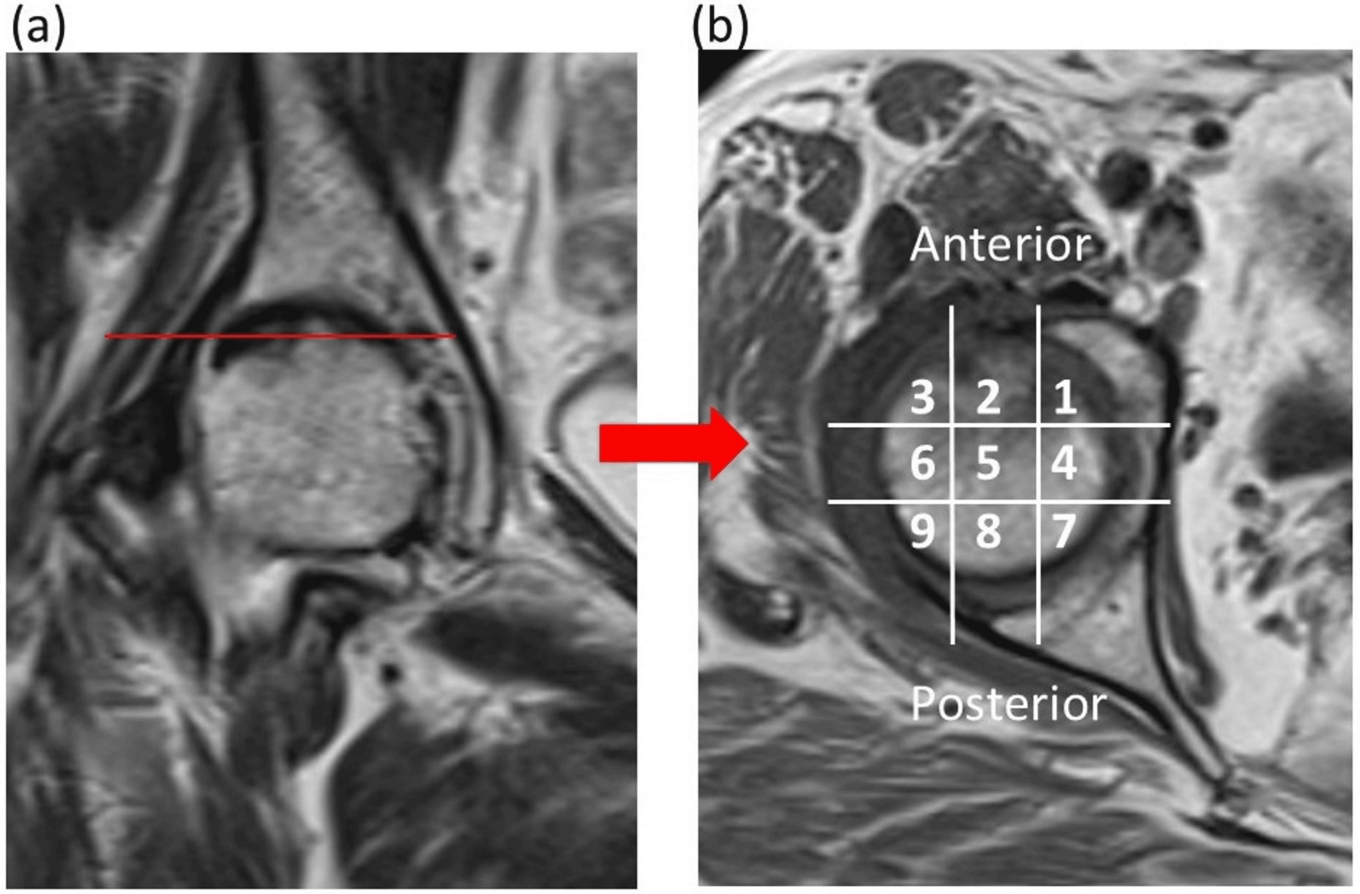
Evaluation of fracture area. (a) T1-weighted coronal view of the largest fracture area. (b) T1-weighted axial view of the fracture line (denoted by the solid red line). The fracture area was evaluated by dividing the femoral head into nine sections in the axial view of the fracture line.

The irregular, very low-intensity band on the T1-weighted coronal MRI view was defined as the fracture line. The slice with the largest fracture area was selected, and the fracture width was measured (Figure 1). The fracture area was evaluated by dividing the femoral head into nine sections in the axial view of the fracture line (Figures 2a, 2b).

**Figure 3:**
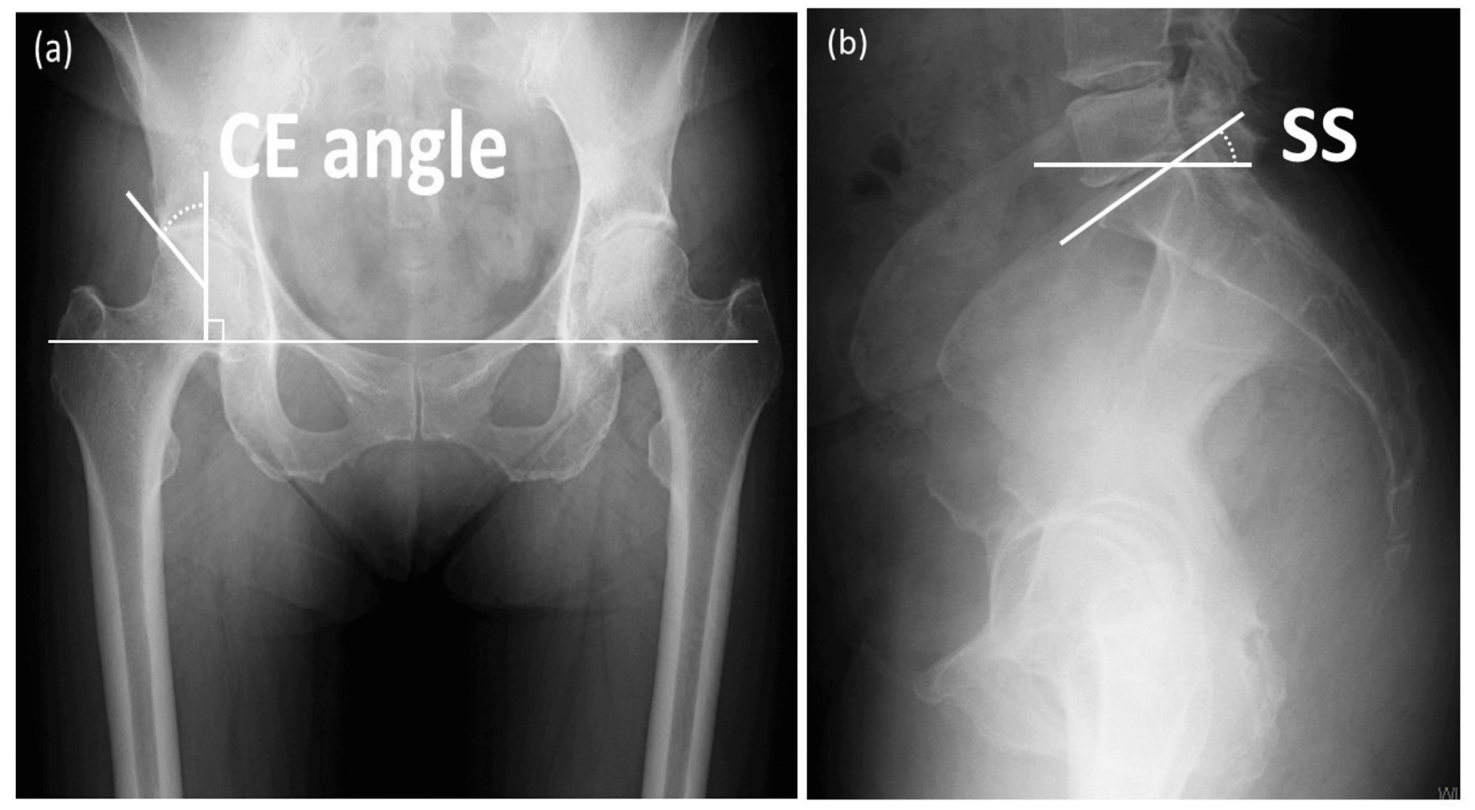
The center edge angle and sacral slope measurement methods. (a) The center edge (CE) angle was measured on anteroposterior radiographs of the hip joint. The CE angle was defined as the angle formed by a vertical line through the center of the femoral head and a line connecting the center of the femoral head and the lateral edge of the acetabulum. (b) Sacral slope (SS) in the standing position were measured on mediolateral radiographs of the hip joint. SS was defined as the angle formed by the S1 endplate and a horizontal line.

Fractures extending into the anterior one-third area of the femoral head (areas 1-3) were considered the anterior (A) group, and those not extending into the anterior one-third were considered the non-anterior (NA) group. The center edge (CE) angle in the supine and standing positions, and SS in the standing position, were measured on anteroposterior (AP) and mediolateral (ML) radiographs of the hip joint (Figures 3a, 3b).

The SS in the supine position was measured using an MRI image or a multiplanar CT reconstruction image of the spinopelvic sagittal view. The CE angle was defined as the angle formed by a vertical line through the center of the femoral head and a line connecting the center of the femoral head and the lateral edge of the acetabulum. SS was defined as the angle formed by the S1 endplate and a horizontal line. Negative values of standing SS - supine SS (ΔSS) were defined as standing posterior pelvic tilt.

Although measurement of the vertical-center-anterior (VCA) angle in the false profile view is the preferred method of evaluating anterior acetabular coverage, only a few patients in this study had a false profile view taken at the time of initial examination. Pelvic reclination of 10° will lead to functional anteversion of the acetabular cup of approximately 7° and functional inclination of approximately 3° [6,7]. Therefore, based on the consideration that there is a correlation between anterior acetabular coverage and external acetabular coverage, anterior acetabular coverage was assessed by the CE angle, which could be measured in all patients in this study.

Patients who had more than 2 mm of collapse of the femoral head from the initial visit to the final follow-up were defined as having progressive collapse of the femoral head. Patients with loss of joint line, in addition to more than 2 mm of collapse of the femoral head, were defined as rapid destructive arthrosis (RDA).

Statistical analysis

Data are expressed as the mean±standard deviation. The Mann-Whitney U test and Fisher's exact probability test were used to compare groups A and NA. P<0.05 was considered significant. All statistical analyses were performed using Statcel (4th Edition) (Tokyo, Japan: OMS, Inc.) - The Useful Add-in Forms on Excel.

## Results

**Figure 4:**
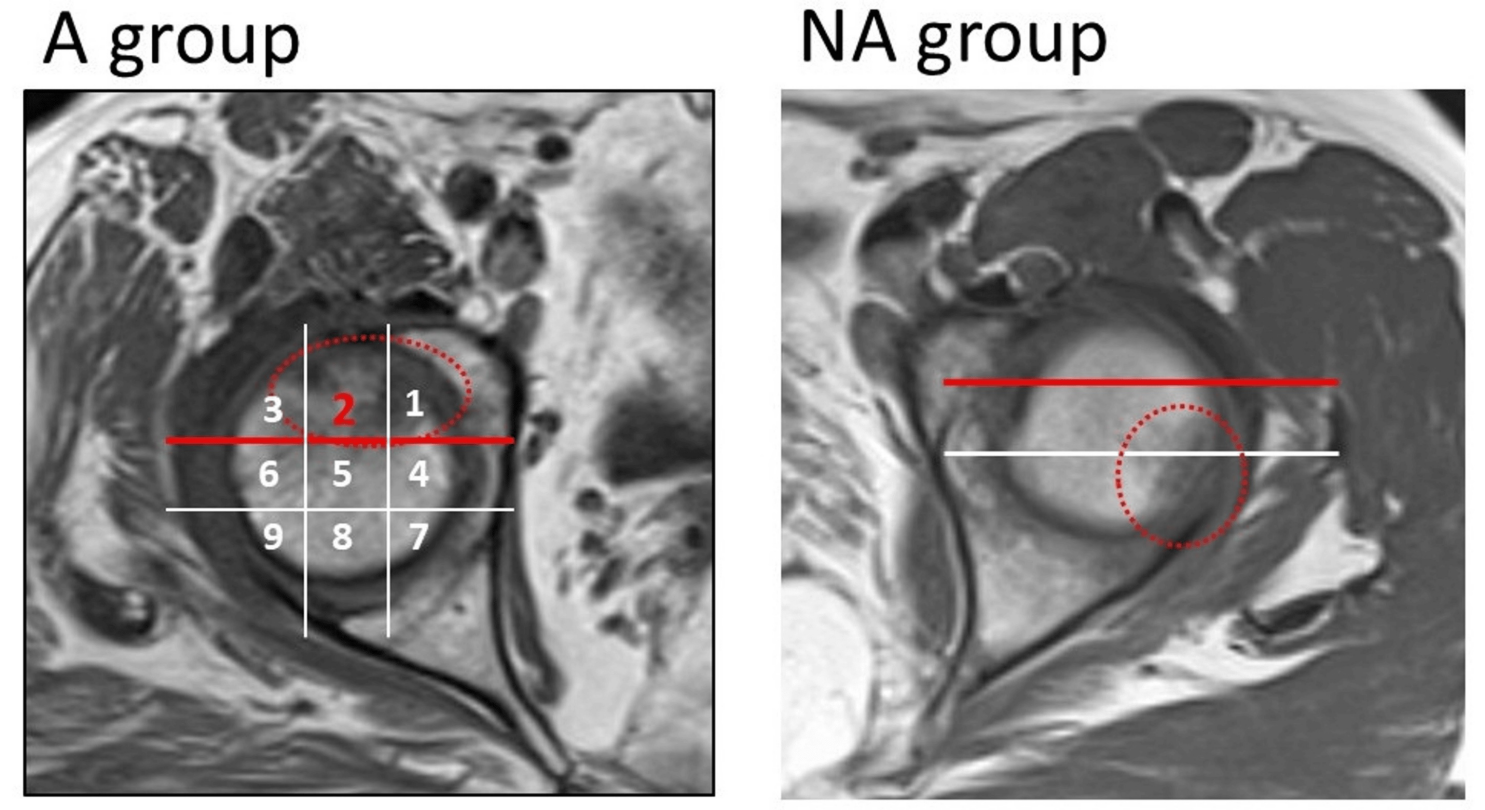
Fracture areas in both groups. Seventeen patients were in the A group and 10 patients were in the NA group. In all cases of the A group, a fracture was found in area 2, which was in contact with the anterior acetabular margin. The A group was defined as fractures extending into the anterior one-third of the femoral head (areas 1-3). The NA group was defined as having no fracture in the anterior one-third of the femoral head. Dotted circle indicates the fracture area. NA: non-anterior; A: anterior

The mean follow-up period was 6.8±4.6 weeks (range: 1-19 weeks). Seventeen patients were in the A group (one male and 16 females, mean age: 73.4±7.2 years, range: 61-89 years), and 10 patients were in the NA group (five males and five females, mean age: 59.1±11.4 years, range: 41-81 years). In all patients in the A group, a fracture was found in area 2, which was in contact with the anterior acetabular margin (Figure 4).

The A group had a higher proportion of females, and they were significantly older (p<0.01) (Table 1). Fracture widths averaged 23.6±7.3 mm (range: 12.7-38.0) in the A group and 13.2±3.6 mm (range: 7.5-18.4) in the NA group (p<0.01). The standing SS - supine SS (ΔSS) averaged -8.6±4.4° (range: -15.4 to -2.6) in the A group and -4.1±1.9° (range: -6.7 to -1.1) in the NA group (p<0.01). The standing CE angle - supine CE angle (ΔCE) averaged -7.1±4.0° (range: -18.6 to -2.1) in the A group and -2.8±2.3° (range: -6.3 to -0.1) in the NA group (p=0.01). Both negative values were significantly greater in the A group, indicating a stronger posterior pelvic tilt in the standing position and reduced acetabular coverage of the femoral head in the standing position.

**Table 1:** Groups and measurements. *Mann-Whitney U test. **Fisher's exact probability test. Values are presented as mean±standard deviation (range). Fractures extending into the anterior one-third area of the femoral head (areas 1-3) were considered the anterior (A) group, and those not extending into the anterior one-third were considered the non-anterior (NA) group. SS: sacral slope; CE: center edge; ΔCE: difference between the standing CE and the supine CE; ΔSS: difference between the standing SS and the supine SS; NA: non-anterior; A: anterior

Variables	A group (n=17)	NA group (n=10)	p-Value
Gender (male:female)	1:16	5:5	<0.01**
Age (years)	73.4 (61 to 89)	59.1 (41 to 81)	<0.01*
Fracture widths (mm)	23.6±7.3 (12.7 to 38.0)	13.2±3.6 (7.5 to 18.4)	<0.01*
ΔSS (°)	-8.6±4.4 (-15.2 to -2.6)	-4.1±1.9 (-6.7 to -1.1)	<0.01*
ΔCE (°)	-7.1±4.0 (-18.6 to -2.1)	-2.8±2.3 (-6.3 to -0.1)	0.01*
Standing CE (°)	27.6±9.1（4.5 to 45.0）	31.1±6.4 (20.3 to 42.9)	0.48*
Supine CE (°)	34.7±7.5 (23.0 to 52.5)	33.9±6.6 (26.6 to 49.2)	0.65*
Progressive collapse, n (%)	11 (65%)	0 (0%)	0.0009**

The ΔCE of the contralateral hip joint averaged -4.6±1.7° (range: -7.3 to -1.8) in the A group and -1.3±0.8° (range: -2.4 to -0.1) in the NA group (p=0.02). The ΔCE comparisons between the affected and the contralateral sides showed no significant differences in either A or NA group (p=0.07, p=0.29). In the A group, there were 11 patients (65%) with progressive collapse of the femoral head, three of whom had RDA. In the NA group, there were no cases of collapse of the femoral head (Table 1).

**Figure 5:**
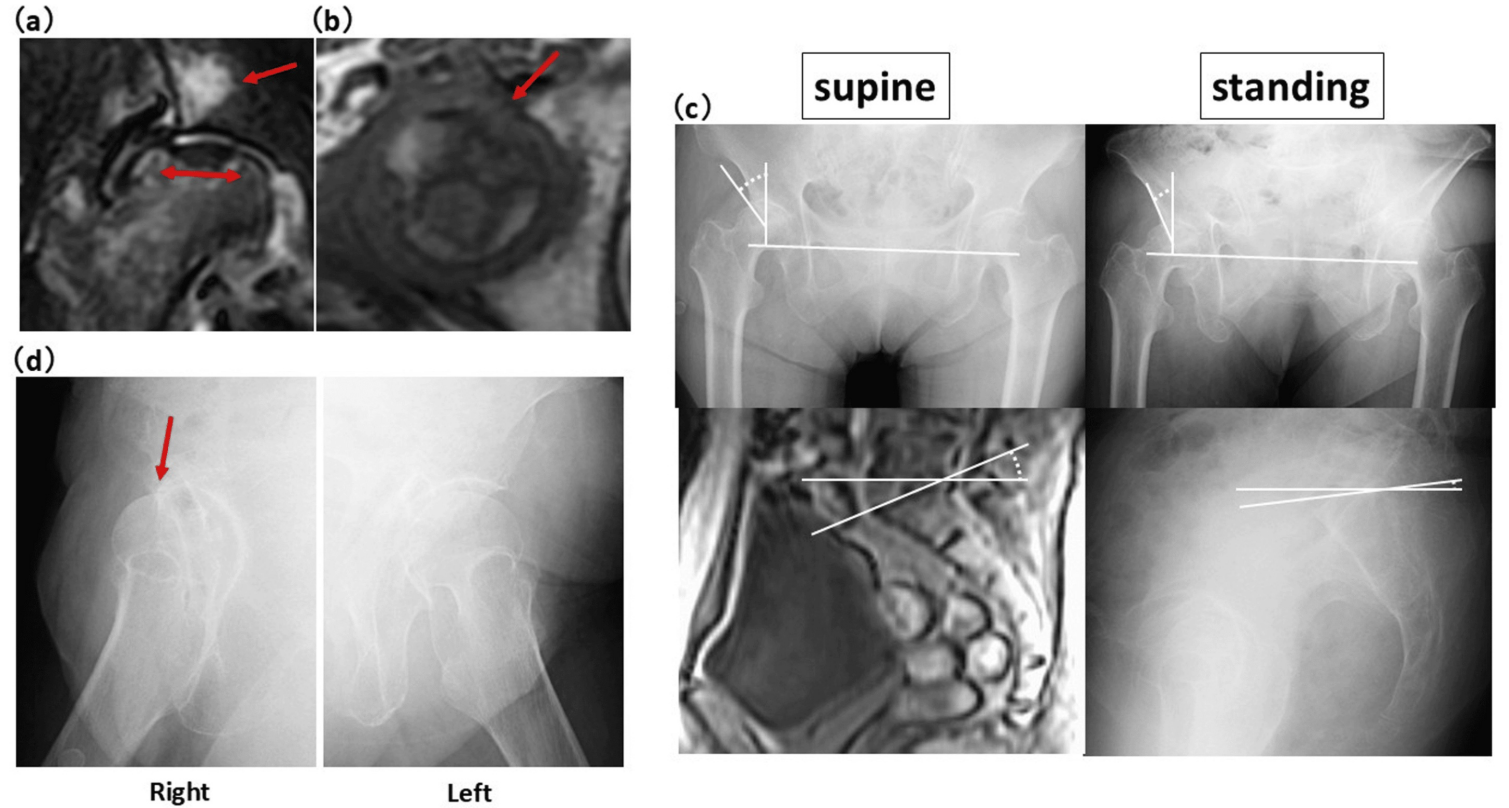
Case 1 - group A. MRI and X-ray images showing a subchondral fracture involving the anterior femoral head. (a) T2-weighted coronal MRI showed a fracture with a maximum width of 26 mm (double-headed arrow), with edematous changes in the anterior-superior portion of the acetabulum (arrow). (b) T1-weighted axial MRI showed a fracture from the anterior superior to the apex of the femoral head, with an anterior superior depression of the femoral head (arrow). (c) CE angle was measured on anteroposterior radiographs of the hip joint. CE angle in the supine position was 33.8° and CE angle in the standing position was 23.3°, a 10.5° decrease in CE angle from supine to standing. SS in the supine position was measured using an MRI image of the spinopelvic sagittal view. SS in the standing position was measured on mediolateral radiographs of the hip joint. SS in the supine position was 21.5° and SS in the standing position was 6.0°, a 15.5° posterior pelvic tilt from supine to standing. (d) False profile view. The anterior coverage of the right acetabulum was small, resulting in a stress concentration on the anterior superior portion of the femoral head (arrow). This case had severe pain and difficulty walking, leading to THA two months after onset. CE: center edge; SS: sacral slope; THA: total hip arthroplasty; A: anterior

**Figure 6:**
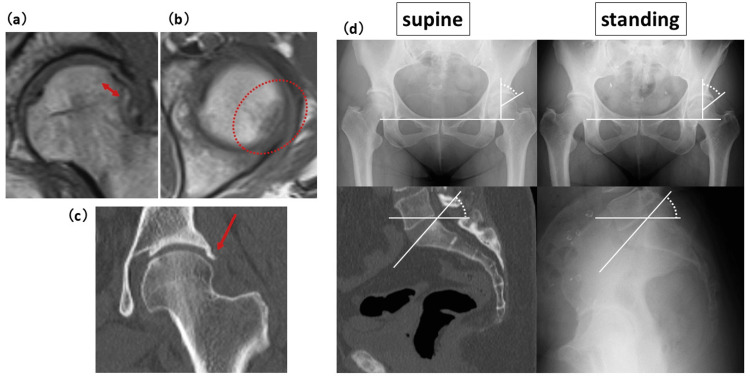
Case 2 - group NA. MRI, CT, and X-ray images showing a subchondral fracture of the posterolateral femoral head. (a) T1-weighted coronal MRI showed a fracture with a width of 7.7 mm (double-headed arrow). (b) T1-weighted axial MRI showed a fracture in the posterolateral area of the femoral head (dotted circle). (c) Coronal view of CT reconstruction image revealed ossification of the acetabular labrum (arrow). (d) CE angle was measured on anteroposterior radiographs of the hip joint. CE angle in the supine position was 49.2° and CE angle in the standing position was 42.9°, a mild decrease of 6.3° in CE angle from supine to standing. SS in the supine position was measured using a multiplanar CT reconstruction image of the spinopelvic sagittal view. SS in the standing position was measured on mediolateral radiographs of the hip joint. SS in the supine position was 48.5° and SS in the standing position was 42.9°, indicating a mild posterior pelvic tilt of 5.7° from supine to standing. CE: center edge; SS: sacral slope; NA: non-anterior

**Figure 7:**
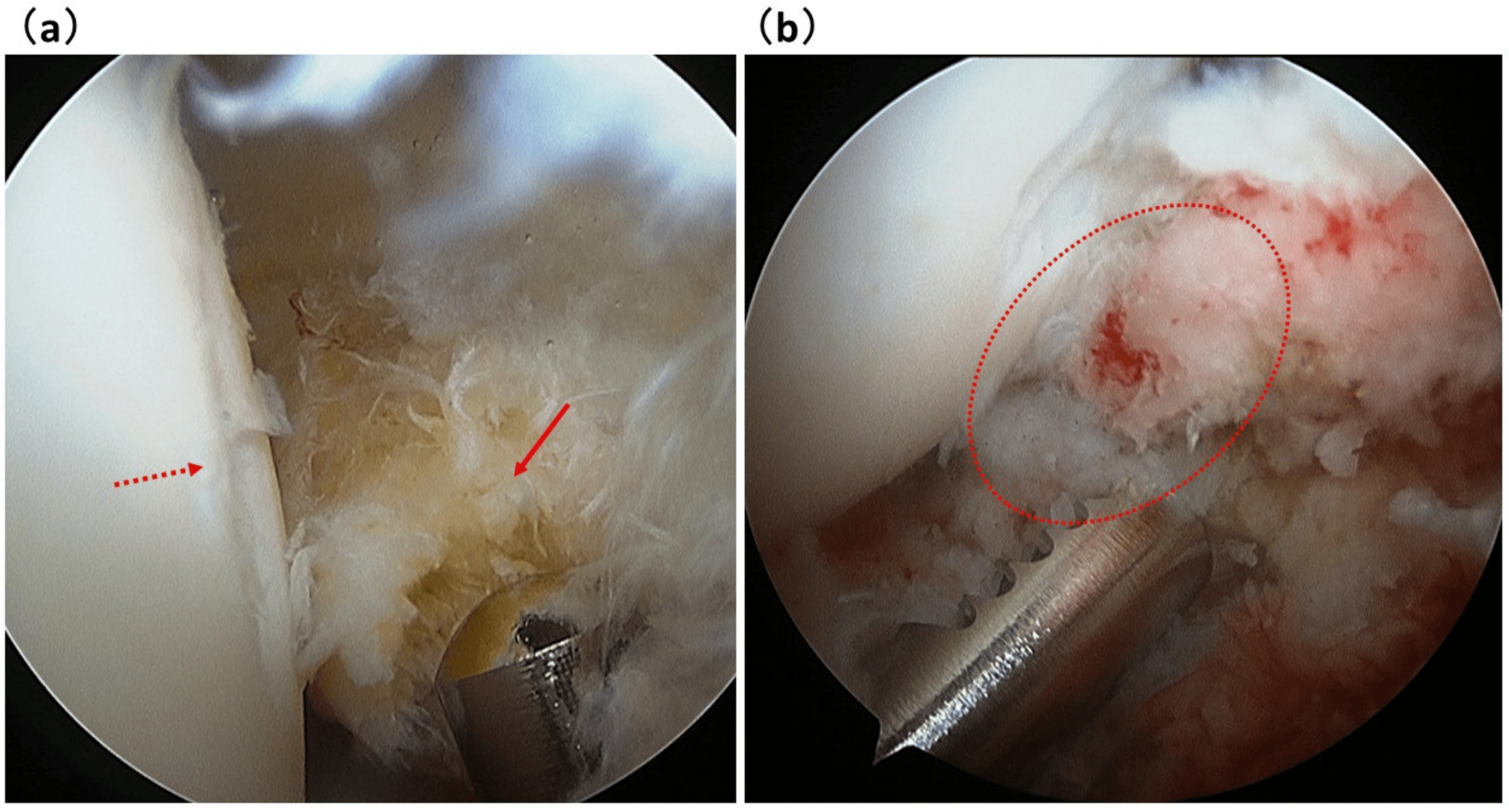
Arthroscopic findings of Case 2. (a) Ossification of the posterolateral acetabular labrum (arrow) and a depression of the posterolateral area of the femoral head that abuts the ossification (dotted arrow). (b) Pain was relieved by arthroscopic excision of the osteophyte (dotted circle). This case is considered to have been a stress fracture caused by impingement.

In group A, 13 patients (76%) received a total hip arthroplasty (THA), with a mean time from onset to THA of 16.6±10.0 weeks (five to 36 weeks). In group NA, joint preservation was possible in half of the patients (Table 2 and Figures 5a-5d, 6a-6d, 7a, 7b).

**Table 2:** Details of treatment in both groups. *Partial excision of the articular labrum. **Excision of osteophytes. Fractures extending into the anterior one-third area of the femoral head (areas 1-3) were considered the anterior group (A group), and those not extending into the anterior one-third were considered the non-anterior group (NA group). THA: total hip arthroplasty; NA: non-anterior; A: anterior

Variables	A group (n=17)	NA group (n=10)
Conservative, n (%)	2 (12%)	3 (30%)
Arthroscopic surgery, n (%)	2 (12%)*	2 (20%)**
THA, n (%)	13 (76%)	5 (50%)

**Figure 8:**
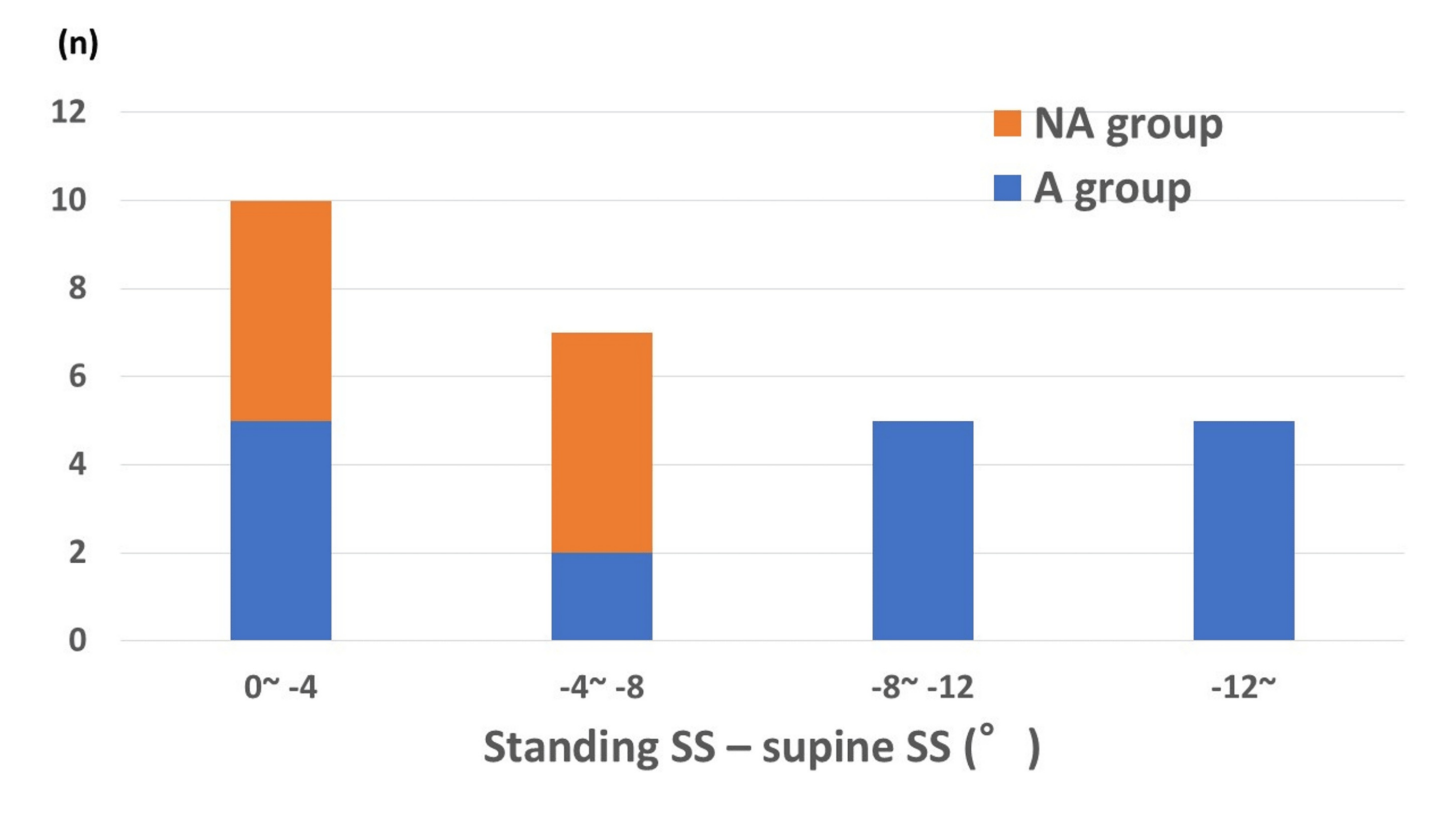
Fracture area and posterior pelvic tilt. The x-axis represents the posterior pelvic tilt. Negative values for standing SS - supine SS (ΔSS) indicate a posterior pelvic tilt in the standing position. The y-axis represents the number of patients. Fractures extending into the anterior one-third area of the femoral head (areas 1-3) were considered the anterior (A) group, and those not extending into the anterior one-third were considered the non-anterior (NA) group. Seventeen patients were in the A group and 10 patients were in the NA group. In patients with a posterior pelvic tilt more than 8° in the supine to standing position, the fracture area was located anteriorly in all cases. SS: sacral slope

In patients with a posterior pelvic tilt of more than 8° in the supine to standing position, the fracture area was located anteriorly in all cases (Figure 8).

## Discussion

In the present study, patients with greater posterior pelvic tilt in the standing position tended to have fractures centered in the anterior superior part of the femoral head, area 2, which is in contact with the anterior acetabular rim, with a greater fracture width and a tendency to collapse. Particularly, in all cases with more than 8° of posterior pelvic tilt in the supine to standing position, the fracture area was located anteriorly. On the other hand, in cases with fractures other than anterior, the posterior pelvic tilt in the standing position was minimal, the fracture width was narrow, and the fracture did not result in collapse. These results indicate that the fracture area differs according to the degree of posterior pelvic tilt in the standing position, and the prognosis also differs.

Subchondral insufficiency fracture (SIF) of the femoral head tends to occur more frequently in elderly women because of bone fragility caused by osteoporosis [8,9]. In a retrospective study of 11 cases of subchondral insufficiency fracture of the femoral head confirmed by histopathology, Yamamoto and Bullough reported that a subchondral insufficiency fracture of the femoral head due to bone fragility can cause rapidly destructive arthrosis of the hip joint (RDA) [1]. Progressive collapse of the femoral head requires surgical treatment. On the other hand, if the onset is early and the collapse is mild, conservative treatments, such as rest and non-weight-bearing, should be tried; however, there is no certainty as to which cases will develop collapse and which will be cured by conservative therapy [10].

There are many reports examining prognostic factors for SIF, and there is a report that low-signal band length on MRI was associated with the progression of collapse of the femoral head [3,11]. Yamamoto et al. reported that SIF was more common in women, and Ikemura et al. reported that bone marrow edema on both acetabular and femoral sides on the MRI image was associated with progression of collapse of the femoral head [12,13]. However, there is still no report on the relationship between posterior pelvic tilt and the prognosis for SIF.

On the other hand, some subchondral fractures of the femoral head have an RDA-like process, and it has been suggested that posterior pelvic tilt is a factor in the development of RDA [1-3]. In a retrospective study of 41 patients with rapid progressive osteoarthritis of the hip, Yasuda et al. reported increased posterior pelvic tilt in a group with collapse of the femoral head within 12 months of the onset of hip pain [4]. Onishi et al. reported that in a study of 34 RDA patients, a pelvic tilt >30° was correlated with a greater degree of femoral head destruction [5].

It has been reported that spinopelvic alignment, so-called spinopelvic harmony, breaks down with age [14,15]. Yukawa et al. reported in a cross-sectional study of healthy Asians that spinopelvic alignment begins to fail rapidly after the age of 70 years, and the pelvis begins to tilt posteriorly, which was more pronounced in women [16]. In this study, the proportion of women tended to be higher in the anterior group, and the mean age was significantly older. This suggests that age-related posterior pelvic tilt in the standing position may be a trigger for the occurrence of subchondral fractures in the anterior superior portion of the femoral head.

In other words, the increasing posterior pelvic tilt in the standing position with aging causes a decrease in the anterior acetabular coverage of the femoral head and a stress concentration on the anterior superior portion of the femoral head during loading, which, combined with age-related bone fragility, results in a significant risk of fracture. This is one of the causes of subchondral insufficiency fracture of the femoral head. On the other hand, there are also subchondral fractures that may have occurred due to local impingement or stress concentrations on the femoral head from ossification of the acetabular labrum or osteophyte formation. In the latter case, if the collapse is mild, joint-preserving therapies, such as conservative treatment with unloading or arthroscopic surgery, should be tried. However, the former is prone to progressive collapse, so total hip arthroplasty (THA) is appropriate for early pain relief and improvement of walking function.

A limitation of this study is that it was based on only 27 patients who had a preserved joint line at the time of initial examination and for whom MRI and standing radiographs of the hip joint were available. Further studies with many more cases are needed. Additionally, if posterior pelvic tilt in the standing position affects femoral head coverage and fracture occurrence, then SIF would be expected to occur bilaterally. However, we could not prove this in the present study. Although we have experience with cases of bilateral SIF or RDA in our department, we have not been able to perform early imaging evaluation, especially MRI evaluation of the fracture area bilaterally, in the early stage of the disease, when the femoral head is not collapsed and the joint line is preserved. Furthermore, the ΔSS threshold used in this study is exploratory and requires validation in a large cohort. However, the finding that posterior pelvic tilt in the standing position is associated with the occurrence of subchondral fractures of the anterior superior femoral head and subsequent collapse may contribute to understanding the pathogenesis of SIF and determining treatment strategies in the future.

## Conclusions

In subchondral fractures of the femoral head, there is an association between fracture area and posterior pelvic tilt in the standing position. Age-related posterior pelvic tilt in the standing position may be a trigger for the occurrence of subchondral fractures in the anterior superior portion of the femoral head. Patients with more than 8° of posterior pelvic tilt in the supine to standing position have a poor prognosis with significant fractures in the anterior superior portion of the femoral head and a tendency toward progressive collapse.

## References

[1] Yamamoto T, Bullough PG (2000). The role of subchondral insufficiency fracture in rapid destruction of the hip joint: a preliminary report. Arthritis Rheum.

[2] Nishida K, Yamamoto T, Motomura G, Shuto T, Nakashima Y, Jingushi S, Iwamoto Y (2005). Early MRI findings of the acetabulum and femoral head in a dysplastic hip resulting in a rapid destruction of the hip joint. Arch Orthop Trauma Surg.

[3] Iwasaki K, Yamamoto T, Motomura G, Ikemura S, Mawatari T, Nakashima Y, Iwamoto Y (2012). Prognostic factors associated with a subchondral insufficiency fracture of the femoral head. Br J Radiol.

[4] Yasuda T, Matsunaga K, Hashimura T (2020). Characterization of rapidly progressive osteoarthritis of the hip in its early stage. Eur J Rheumatol.

[5] Onishi E, Ota S, Fujita S (2022). Association between sagittal spinopelvic alignment and femoral head destruction in the early stage of rapidly destructive coxopathy. Bone Jt Open.

[6] Lembeck B, Mueller O, Reize P, Wuelker N (2005). Pelvic tilt makes acetabular cup navigation inaccurate. Acta Orthop.

[7] Kanawade V, Dorr LD, Wan Z (2014). Predictability of acetabular component angular change with postural shift from standing to sitting position. J Bone Joint Surg Am.

[8] Bangil M, Soubrier M, Dubost JJ, Rami S, Carcanagues Y, Ristori JM, Bussiere JL (1996). Subchondral insufficiency fracture of the femoral head. Rev Rhum Engl Ed.

[9] Yamamoto T, Bullough PG (1999). Subchondral insufficiency fracture of the femoral head: a differential diagnosis in acute onset of coxarthrosis in the elderly. Arthritis Rheum.

[10] Yamamoto T (2012). Subchondral insufficiency fractures of the femoral head. Clin Orthop.

[11] Hackney LA, Lee MH, Joseph GB, Vail TP, Link TM (2016). Subchondral insufficiency fractures of the femoral head: associated imaging findings and predictors of clinical progression. Eur Radiol.

[12] Yamamoto T, Karasuyama K, Iwasaki K, Doi T, Iwamoto Y (2014). Subchondral insufficiency fracture of the femoral head in males. Arch Orthop Trauma Surg.

[13] Ikemura S, Mawatari T, Matsui G, Iguchi T, Mitsuyasu H (2016). Clinical outcomes in relation to locations of bone marrow edema lesions in patients with a subchondral insufficiency fracture of the hip: a review of fifteen cases. Br J Radiol.

[14] Schwab F, Lafage V, Boyce R, Skalli W, Farcy JP (2006). Gravity line analysis in adult volunteers: age-related correlation with spinal parameters, pelvic parameters, and foot position. Spine (Phila Pa 1976).

[15] Schwab F, Patel A, Ungar B, Farcy JP, Lafage V (2010). Adult spinal deformity - postoperative standing imbalance: how much can you tolerate? An overview of key parameters in assessing alignment and planning corrective surgery. Spine (Phila Pa 1976).

[16] Yukawa Y, Kato F, Suda K, Yamagata M, Ueta T, Yoshida M (2018). Normative data for parameters of sagittal spinal alignment in healthy subjects: an analysis of gender specific differences and changes with aging in 626 asymptomatic individuals. Eur Spine J.

